# PANoptosis in neurological disorders: mechanisms, implications, and therapeutic potential

**DOI:** 10.3389/fimmu.2025.1579360

**Published:** 2025-06-11

**Authors:** Jingyi Li, Yi Qu

**Affiliations:** ^1^ Department of Pediatrics, West China Second University Hospital, Sichuan University, Chengdu, Sichuan, China; ^2^ Key Laboratory of Birth Defects and Related Diseases of Women and Children, Sichuan University, Ministry of Education, Chengdu, Sichuan, China

**Keywords:** PANoptosis, PANoptosome, Ninj1, central nervous system disorders, inflammation

## Abstract

PANoptosis is a unique form of programmed cell death (PCD) that has the combined main features of pyroptosis, apoptosis, and necroptosis, but cannot be fully explained by any single pathway alone. Among all the influencing factors in PANoptosis, the activation and assembly of PANoptosomes are the most critical. To date, four distinct PANoptosomes have been identified: Z-DNA-binding protein 1 (ZBP1), absent in melanoma 2 (AIM2), receptor-interacting protein kinase 1 (RIPK1), and nucleotide-binding leucine-rich repeat-containing receptor 12 (NLRP12) PANoptosomes. Currently, PANoptosis is a promising target for central nervous system (CNS) disorders treatment. Understanding its mechanisms will facilitate its therapeutic application. This review introduces the concept of PANoptosis, its detection methods, the molecular composition and regulation of PANoptosomes, and the role of ninjurin 1 (NINJ1), a new “executor” in PANoptosis. In addition, recent therapeutic advances targeting PANoptosis in CNS diseases were also discussed. Future research on inhibiting PANoptosis, the dynamic regulatory relationships among three death pathways, and the interactions with NINJ1 will offer new clinical insights.

## 1 Introduction

As a biologically active mechanism, PCD plays a crucial role in organismal development and serves as a defence strategy against infectious factors (1). Among the various forms of PCDs, pyroptosis, apoptosis, and necroptosis are the most extensively studied (2). Pyroptosis, an inflammasome-mediated process, is characterised by the formation of pores on the plasma membrane depending on caspase-1 (CASP1) (2, 3). Apoptosis is a conserved form of PCD initiated by extrinsic and intrinsic signaling pathways, identified by a series of distinct morphological alterations like cellular retraction, chromatin condensation, DNA fragmentation, and apoptotic body formation while keeping the plasma membrane intact (4). Necroptosis is mainly mediated by RIPK3 and is marked by cell swelling and loss of membrane integrity. Studies have shown that when CASP8 is inhibited, proteins including RIPK1, RIPK3, mixed lineage kinase domain-like protein (MLKL), Fas-associated via death domain (FADD), and procaspase-8 can form complex IIb, resulting in necroptosis (5).

These three PCDs have historically been described as independent signaling pathways. However, accumulating evidence indicates widespread crosstalk between them, forming the concept of PANoptosis. The PANoptosome complex is essential for PANoptosis, just as inflammasomes for pyroptosis, apoptotic complex II for apoptosis, and necrosomes for necroptosis (6). The composition of PANoptosome varies with the trigger but typically includes ZBP1, AIM2, RIPK3, RIPK1, apoptosis-associated speck-like protein (ASC), FADD, CASP8, and other key components for these three PCDs (1).

PANoptosis activation elicits a strong immune response, but abnormal activation may cause excessive inflammation, cytokine storm, and issue and organ damage subsequently (6). Numerous studies indicate that PANoptosis is significantly associated with the progression of immune diseases, neurological disorders, and cancer. While research on PANoptosis in CNS diseases is limited, many studies have highlighted the key roles of neuroinflammation and cell death in disease progression, suggesting PANoptosis involvement. During diseases, pathogen-associated molecular patterns (PAMPs) or damage-associated molecular patterns (DAMPs) are recognized by pattern recognition receptors (PRRs), thereby initiating downstream signaling cascades that produce inflammatory factors, activate inflammasomes, and potentially trigger multiple cell death pathways simultaneously, leading to irreversible neuronal death (7). For instance, the AIM2-PANoptosome is involved in Alzheimer’s disease (AD), as AIM2 knockout suppresses key AD-related events like Aβ-deposition and microglial activation (8, 9). Yan et al. utilized bibliometric analysis and data mining to hypothesize that PANoptosis contributes to brain ischemia/reperfusion (I/R) injury, which has been verified (10); In sepsis-associated encephalopathy (SAE), researchers first confirmed the PANoptosis involvement, and then revealed that the synergistic effect between apoptosis and pyroptosis modulates necroptosis, highlighting the crosstalk among these PCDs (11). Based on the current evidence, we speculate that PANoptosis and PANoposome also play significant roles in CNS disease pathogenesis.

In this review, we summarise the discovery and detection methods of PANoptosis and PANoptosomes, the composition of different types of PANoptosomes, and the role of ninjurin 1 (NINJ1). We also delve into the implication and mechanism of PANoptosis in CNS disorders and finally discuss the potential of therapeutic strategies targeting PANoptosis in CNS disorders.

## 2 The discovery of PANoptosis and PANoptosome

Kuriakose et al. reported the first case of PANoptosis. They revealed that macrophages with influenza A virus (IAV) infection undergo pyroptosis, apoptosis, and necroptosis concurrently, characterized by CASP1/3/8 activation and MLKL phosphorylation (12). The concept of PANoptosis was first proposed by Kanneganti et al. in 2019 (1, 2, 6, 13, 14). PANoptosis was initially defined as a combination of the main features of pyroptosis, apoptosis, and necroptosis (1). A series of studies on PANoptosis by Kanneganti et al. showed that diseases resulting from pathogens like bacteria and viruses can induce an autoimmune response and trigger inflammatory cytokines production, which then triggers the promoter proteins and ultimately leads to PANoptosome formation. The concept of PANoptosome was first proposed by Christian et al. in 2020. They discovered that molecules involved in pyroptosis, apoptosis, and necroptosis interact to form the PANoptosome complex (12, 13, 15–19). Since then, more types of PANoptosomes have been identified. Proteins that make up PANoptosome can be categorized into three main groups: 1) pathogen‐associated molecular patterns (PAMPs) or damage‐associated molecular patterns (DAMPs) sensors, such as ZBP1, AIM2, NLRP3; 2) adaptors, such as ASC and FADD; 3) catalytic effectors, like RIPK1, RIPK3, CASP1, and CASP8 (1, 2).

Consequently, PANoptosis is an inflammatory PCD pathway that is activated by specific triggers and regulated by PANoptosomes. It exhibits key features of pyroptosis, apoptosis, and/or necroptosis, yet cannot fully account for any single one (1, 2).

The concept of PANoptosis was developed based on pathogen infection models. Considering the involvement of cell death and inflammation in CNS diseases, researchers have hypothesized that PANoptosis may play a role in CNS disorders and validated this hypothesis by experiments. Yan et al. analyzed the experimental data supporting PANoptosis presence in the I/R, which is characterized by a significant upregulation of key proteins in death pathways. Furthermore, the expression of the PANoptosome components, such as NLRP3, CASP-1, ASC, CASP-8, RIPK1, and RIPK3, were increased, while some of those were found to interfere with two or three death pathways simultaneously. Based on these findings, the authors speculated that the PANoptosome might play a key role in the I/R process (10). In addition, Yetirajam et al. summarized the currently known molecules involved in PANoptosis in AD, which are linked to three PCDs and multiple inflammasomes (9). They also suggested that the AIM2-PANoptosome might be implicated in AD pathogenesis, as AIM2 deficiency reduced Aβ accumulation and microglial activation in the 5xFAD mouse model (8). An increasing number of researchers are applying the concept of PANoptosis to CNS diseases, aiming to regulate interactions among the three PCDs to change cell viability, mitigate brain tissue inflammatory responses, and ultimately improve clinical neurological outcomes.

## 3 The regulation of PANoptosis

### 3.1 Types of PANoptosome

PANoptosome serves as a molecular scaffold for binding key PANoptotic molecules (1, 2, 14). Various types of PANoptosomes have been characterized, including ZBP1, AIM2, RIPK1, and NLRP12. In addition, NLRP3 and NLRC4 inflammasomes have received significant attention because they play critical roles in PANoptosis. To date, many studies have focused on factors regulating the synthesis and assembly of PANoptosome components, providing a theoretical basis for future therapeutic interventions.

#### 3.1.1 ZBP1-PANoptosome and its regulation in PANoptosis

**Figure 1:**
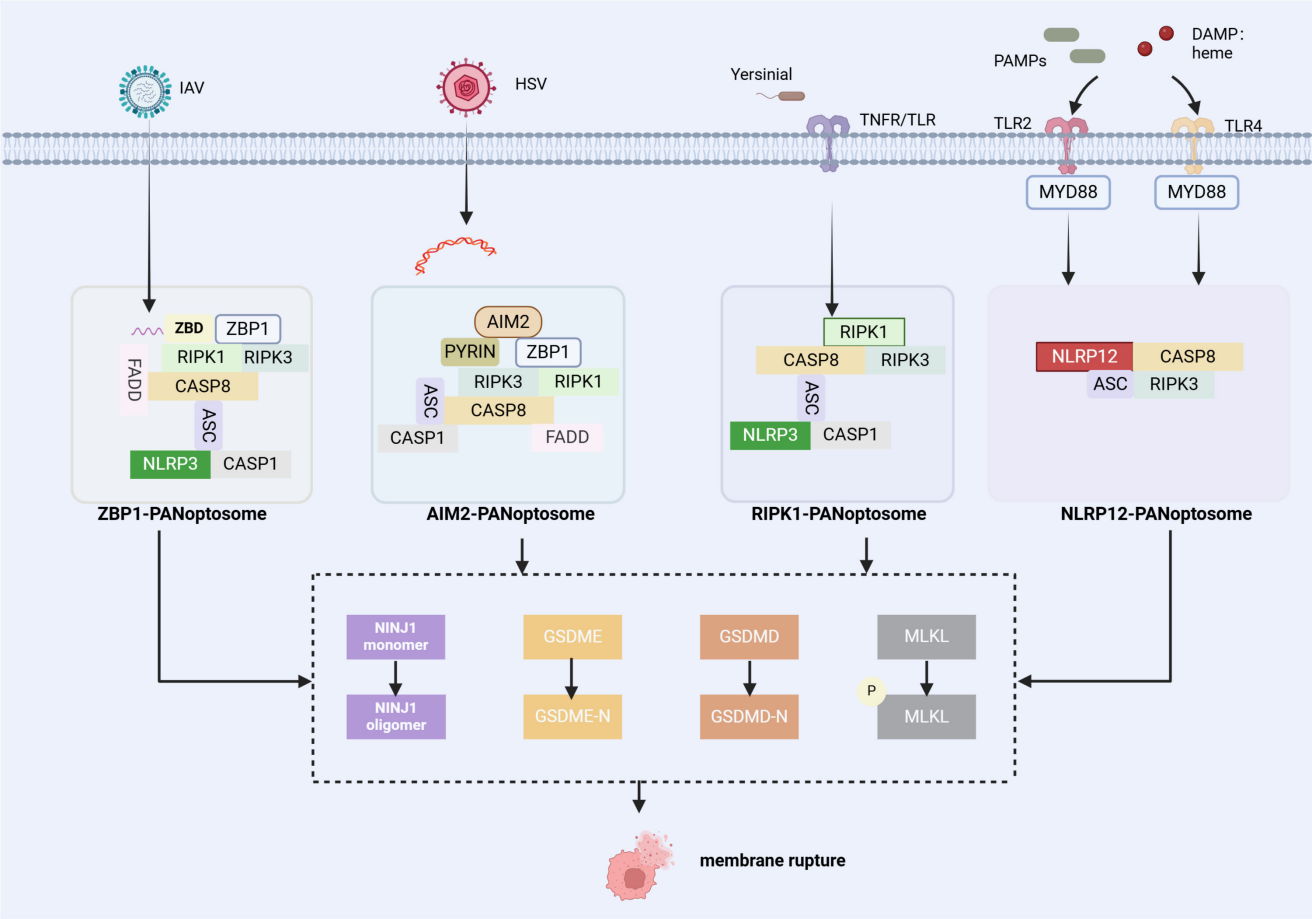
Summary of the assembly of PANoptosome complex. (1) The ZBP1 PANoptosome consists of ZBP1 (a sensor protein), RIPK1 and RIPK3 (proteins involved in necroptosis), FADD and CASP8 (proteins associated with apoptosis), and NLRP3, ASC, and CASP1 (proteins involved in inflammasome/pyroptosis). (2) The AIM2 PANoptosome includes AIM2 (a sensor protein), ZBP1, ASC, CASP1 (proteins in inflammasome/pyroptotic), CASP8 and FADD, and RIPK3 and RIPK1. (3) The RIPK1 PANoptosome includes RIPK1 (a sensor protein), RIPK3, ASC, CASP1 and CASP8. (4) The NLRP12 PANoptosome includes NLRP12 (a sensor protein), ASC, CASP8, and RIPK3. PANoptosomes function as molecular platforms to initiate PANoptosis, and various factors (RED) can modulate the assembly process of PANoptosomes. NINJ1 is a key executor of PANoptosis and functions independently of other pore-forming proteins[The figure was created using BioRender (https://BioRender.com)].

ZBP1, a classic PANoptosome sensor, was first identified during IAV infection. During infection, ZBP1 responds to IAV and forms the ZBP1-RIPK3 complex, which then facilitates recruitment of RIPK1 and further forms ZBP1-PANoptosomes with ZBP1/RIPK3/RIPK1/FADD/CASP8 as the main components (12, 20, 21). The composition of the ZBP1-PANoptosome is shown in **Figure 1**.

ZBP1, initially known as DNA-dependent activator of interferon regulatory factors(DAI), is strongly upregulated by type I IFN signaling (22). Interferon regulatory factor 1 (IRF1) is a transcriptional regulator of ZBP1 and a well-recognised regulator of cell death. During IAV infection, IRF1 exerts an upstream regulatory role by modulating ZBP1 expression and regulating ZBP1-mediated PANoptosis subsequently (23). To date, four proteins have been identified to own a Z-binding domain (ZBD), a 78-amino acid protein fold that specifically recognizes and binds to Z-DNA and Z-RNA to activate ZBP1, while only ADAR1 and ZBP1 among mammalian proteins are involved in innate immune responses (14). Both ADAR1 and ZBP1 contain Zα domains and are IFN-induced, but ADAR1 does not play a similar role in cell death. Karki et al. conducted further investigations into the underlying mechanism. They revealed that ADAR1 inhibits PANoptosis by interacting with the Zα domain of ZBP1 and restricting the ZBP1-RIPK3 binding. Nuclear export inhibitors (NEIs) sequester ADAR1 in the nucleus, limiting its editing functions and enabling ZBP1 to bind to RIPK3 (19). Post-translational modification of ZBP1 also plays a crucial role in regulating PANoptosis. For example, ZBP1 ubiquitination markedly increases following IAV infection (24). In a study on sepsis with lactic acidaemia and acute lung injury, the investigators found that a tripartite motif containing 32 (TRIM32) interacted with ZBP1, accelerating its ubiquitination and degradation. Extracellular cold-inducible RNA-binding protein (eCIRP) blocks this process by competitively binding to ZBP1, thereby stabilizing ZBP1 to mediate PANoptosis (25).

#### 3.1.2 AIM2-PANoptosome

AIM2 is a cytoplasmic innate immune PRR that recognizes pathogens and endogenous dsDNA (26–28). Kari et al. first observed ASC interactions with AIM2, ZBP1, pyrin, CASP1, CASP8, RIPK3, RIPK1, and FADD through immunoprecipitation after Herpes simplex virus (HSV1) and Francisella novicida infections; While 12 hours post-infection, ASC spots co-localized with AIM2, ZBP1and pyrin within the same cells, suggesting AIM2-PANoptosome formation during infection. **Figure 1** shows the composition of this PANoptosome.

Deletion of AIM2 reduced ZBP1 and pyrin expression, indicating AIM2-mediated signaling regulates these proteins upstream. However, the activation of CASP1/GSDMD/GSDME, CASP8/3/7, and RIPK3/MLKL was reduced in *Mefv^–/–^* and *Zbp1^–/–^* bone-marrow-derived macrophage (BMDMs), and completely inactivated in *Mefv* ^–/–^ *Zbp1^–/–^* BMDMs, suggesting that pyrin and ZBP1 synergistically promote AIM2-mediated PANoptosis (28). Sharma et al. showed that cells deficient in IRF1 exhibited decreased *Aim2* mRNA and protein expression upon HSV1 infection. The PANoptosis molecules activation, DAMPs releasing, and cell mortality were correspondingly reduced, suggesting that IRF1 modulates AIM2-mediated PANoptosis by regulating AIM2 expression (29).

#### 3.1.3 RIPK1-PANoptosome

RIPK1 is a key regulator of TNF receptor-1 (TNFR1) signaling and promotes the transcription of intracellular cytokines (30). It plays a crucial role in necroptosis, especially when CASP8 is inhibited (14). Briard et al. showed that Yersinia infection induced RIPK1-PANoptosome complex formation. Notably, this study demonstrated that RIPK1 deficiency did not significantly reduce overall cell death because RIPK1 deficiency decreased pyroptosis and apoptosis but concurrently increased necroptosis (31). **Figure 1** shows its composition.

IRF1, an upstream regulator of PANoptosis, regulates RIPK1-mediated PANoptosis (29). After SARS-CoV-2 infection, TNF-α and IFN-γ jointly induce PANoptosis. Downstream of TNF-α and IFN-γ, the JAK/STAT1 pathway controls the transcriptional regulation of IRF1, which further activates inducible nitric oxide synthase (iNOS) and nitric oxide (NO) production. Subsequently, NO activates PANoptosis through the RIPK1/FADD/CASP8 axis (32). However, in the study of human cancer cells, TNF/IFNγ treatment did not induce iNOS expression or NO production. IRF1 directly binds to the promoters of CYLD and CASP8, activating their transcription. Therefore, the authors proposed that variations in key component expression across different cell lines influence the specific downstream molecules of caspase-8 involved in cell death (33). Post-translational modifications of RIPK1 also modulate RIPK1-mediated PANoptosis. Liu et al. showed that the inhibition of cIAP1/2 could attenuate sepsis-induced inflammatory responses and lung injury by decreasing the ubiquitination and phosphorylation of RIPK1, while they also found the upregulation of apoptosis, necroptosis, and pyroptosis(PANoptosis) (34). Researchers found that transforming growth factor-β-activated kinase 1 (TAK1) inhibits RIPK1 phosphorylation, limiting RIPK1 activation and blocking spontaneous PANoptosis. Conversely, RIPK1 deletion rescues cell death in TAK1-deficient macrophages (13, 35, 36). However, another study found that cells have evolved a mechanism to sense TAK1 inactivation independent of RIPK1 kinase activity. Malireddi et al. revealed that TLR priming could bypass RIPK1 kinase activity and drive PANoptosis in TAK1-deficient cells, indicating that inhibition of RIPK1 kinase activity may not be effective in treating PANoptosis-related diseases (37). The team also conducted a genome-wide clustered regularly interspaced short palindromic repeats (CRISPR) screening of macrophages, which led to the identification of a previously unknown TAK1i-induced regulator of cell death, ribonucleoprotein, PTB binding 1 (RAVER1). In this study, RAVER1 prevented the alternative splicing of RIPK1, and its genetic depletion suppressed the TAK1i-induced RIPK1-mediated inflammasome activation and PANoptosis (38).

#### 3.1.4 NLRC5 and NLRP12-PANoptosome

NLRP12 is a cytoplasmic sensor involved in innate immune response and activates inflammasomes and PANoptosomes. Immunoprecipitation revealed that the multiprotein PANoptosome complex, which contains ASC, RIPK3, CASP8, and NLRP3, was formed in wild-type (WT) BMDMs treated with haem and Pam3 (39). Research has clarified that haem and PAMPs activate TLR2/4, triggering MyD88-mediated signaling pathways that lead to IRF1 expression and ROS production. This upregulates Nlrp12, initiating the formation of PANoptosome complexes and inducing PANoptosis (39–41). The discovery of the NLRP12 PANoptosome provides potential therapeutic approaches for treating haemolytic diseases.

NLRC5, the final member of the NLRC subfamily, is a primary histocompatibility complex class I transcriptional activator. Mutations in the NLRC5 gene are associated with poor prognosis and increased risk of inflammatory and infectious diseases in cancer patients (42). In addition to regulating MHC class I gene expression, NLRC5 can also drive inflammasome activation and interact with NLRP3 to negatively regulate NF-κB and interferon-dependent gene transcription (43). Sundaram et al. found that NLRC5 can respond to PAMPs/DAMPs(including haem) and DAMP/cytokine combinations and form a PANoptosome by interacting with NLRP12 or NLRP3, which includes NLRC5, ASC, RIPK3, CASP8, and NLRP3 (44). Notably, NLRC5 does not regulate CASP1 activation or inflammatory cytokines release, whereas NLRP12 does, highlighting their distinct roles (45).

#### 3.1.5 NLRP3 inflammasome

NLRP3 is the most extensively studied sensor protein and a global sensor for PAMPs and DAMPs (46). NLRP3 activation aids the host in defending against microbial infections, and its dysregulation is associated with various inflammatory diseases.

During IAV infection, ZBP1 plays a key role by detecting Z-RNA, which recruits RIPK3 and CASP8, leading to NLRP3 inflammasome activation (12, 47).During IAV infection, the activation of NLRP3 inflammasome entirely hinges on ZBP1; however, ZBP1 is not essential for activation in response to other RNA viruses such as vesicular stomatitis virus (VSV28) (47). TAK1 is a crucial regulator of innate immunity, inflammation, and cell death. Consequently, several pathogens produce TAK1i. Studies have shown that TAK1 deficiency in macrophages induces spontaneous NLRP3 inflammasome activation without the need for TLR priming or subsequent signalling (35). Zheng et al. found that CASP6 is crucial for ZBP1-mediated NLRP3 inflammasome activation (21). During IAV infection, CASP6 serves as a scaffold that enhances the interaction between RIPK3 and ZBP1, independent of its caspase activity (48).

#### 3.1.6 NLRC4-inflammasome

NLRC4 inflammasomes are primarily activated by specific bacterial components, particularly type III secretion system (T3SS) and flagellar proteins. Neuronal apoptosis inhibitory protein (NAIP) proteins, such as NAIP2 and NAIP5, serve as upstream immunosensors for NLRC4 inflammasome activation (16, 49, 50). Although PANoptosis is most commonly described in conjunction with the activation of NLRP3 inflammasome, evidence suggests that NLRC4 may also serve a pivotal function in this death pathway (16). Previous research indicates that NAIP/NLRC4 inflammasomes activate during Salmonella typhimurium infection, and a recent study suggests they may also play a role in PANoptosis during infection (16, 51). Up to now, the connections between the NLRC4 inflammasome and the three classic death pathways have been reported (52–55). NLRP1b and NLRC4 can trigger CASP8-mediated apoptosis, representing an alternative cell death pathway in macrophages and intestinal epithelial organoids (IECs) that lack CASP1 (53, 54). A study on ischemic stroke found that the activated NLRC4-inflammasome may mediate pyroptosis and apoptosis via CASP1 and CASP8 cleavage (55). During Pseudomonas aeruginosa infection, BMDMs lacking NLRC4 or NAIP5 exhibited increased necroptosis markers RIPK1 and MLKL activation (56). However, evidence for a direct association between the NLRC4 inflammasome and PANoptosis remains insufficient.

The distinction between inflammasomes and PANoptosomes is important. We will use the classic NLRP3 inflammasome to compare the two concepts. In terms of composition, the NLRP3 inflammasome can be part of the PANoptosome in some cases, while the PANoptosome has a more complex structure and integrates elements from multiple cell death pathways. Notably, even for the same PANoptosome, its specific composition and functional characteristics can change dynamically depended on the triggers (such as the species of pathogens). **Table 1** shows the main components of both. Functionally, the NLRP3 inflammasome mainly leads to pyroptosis and the release of IL-1β/IL-18, while the PANoptosome has a broader role: it simultaneously triggers three classic PCDs, accompanied by a stronger inflammatory response and cytokine storm. Notably, only the NLRP3 inflammasome has been proven to assemble the PANoptosome, the evidence for the NLRC4 inflammasome’s role in PANoptosis is still insufficient.

**Table 1:** Comparison of different types of PANoptosomes.

Types of PANoptosome	Main component	Classical triger	Regulatory factor	Change of PANoptosis	Reference
ZBP1-PANoptosome	ZBP1/ASC/CASP1/NLRP3/FADD/CASP8/RIPK3/RIPK1	Influenza A virus	Type I IFN signal: activating IRF1 and upregulates ZBP1 expression.	↑	(23, 32)
			ADAR1:binding to ZBP1 and competitively inhibits the binding of ZBP1 to RIPK3.	↓	(19)
			ZBP1 Ubiquitination:Ubiquitinated ZBP1 is degraded.	↓	(25)
AIM2-PANoptosome	AIM2/ASC/Pyrin/ZBP1/CASP1/FADD/CASP8/RIPK3/RIPK1	HSV1 、 F. novicida	IRF1:upregulating AIM2 expression	↑	(29)
			Pyrin and ZBP1: synergistically promote AIM2-mediated PANoptosis.	↑	(28)
RIPK1-PANoptosome	RIPK1/ASC/CASP1/NLRP3/CASP8/RIPK3	Yersinia	IRF1 :upregulating RIPK1 expression	↑	(29)
			TAK1: Inhibiting the phosphorylation of RIPK1	↓	(13, 35, 36)
			cIAP1/2:Inhibiting the ubiquitination and phosphozrylation of RIPK1	↓	(34)
			RAVER1: blocking the Selective splicing of RIPK1	↓	(38)
NLRC5 and NLRP12-PANoptosome	NLRC5/NLRP12/ASC/RIPK3/CASP8	Heme plus PAMPs	IRF1: upregulating NLRP12 expression	↑	(29)
			TLR signaling and NAD+ levels: activated TLR signaling leads to NAD+ degradation, which activates sirtuins and then promotes the expression of NLRC5.	↑	(44)
NLRP3 inflammasome	NLRP3/ASC/CASP1	The priming signal: microbial components or endogenous cytokines The activation signal: extracellular ATP, pore-forming toxins, or particulate matter	TAK1 deficiency: inducing spontaneous activation of the NLRP3 inflammasome	↑	(35)
			ZBP1: during IAV infection, ZBP1 regulates NLRP3 inflammasome activation via the RIPK1–RIPK3–CASP8 axis	↑	(12)
			CASP6:during IAV infection, CASP6 enhances the interaction between RIPK3 and ZBP1, promoting ZBP1-Mediated NLRP3 Inflammasome Activation	↑	(21, 48)
NLRC4-inflammasome	NLRC4/ASC/CASP1	Bacterial components related to type III secretion system (T3SS) and flagellin	NA	NA	NA

↓: It means PANoptosis is inhibited, (reduced PANoptosis-related expression, lower cell death, etc.); ↑: It means PANoptosis is activated, (increased PANoptosis-related proteins expression and an increase in cell death rate, etc).

In summary, PANoptosomes integrate key molecules from three classic PCDs, exerting both scaffolding and catalytic functions to induce PANoptosis (14). Many other proteins are anticipated to emerge as novel components of PANoptosomes. For instance, the RNA sensor RIG-I is a PRR that detects both ssRNA and dsRNA. It plays a key role in RNA virus infection by forming complexes with the PANoptosome core members, CASP8 and RIPK1, facilitating interferon signalling (65, 66). Researchers also described a complex consisting of FADD, RIPK1, and CASP8 that is activated by toll/IL-1R domain-containing adaptor-inducing IFN-β (TRIF) signalling, termed “TRIFosome.” When TAK1 is inhibited, this complex strongly associates with lipopolysaccharide (LPS)-induced cell death (67). More research is needed to understand how these protein complexes are involved in PANoptosis.

### 3.2 The role of NINJ1 protein in PANoptosis

NINJ1, a transmembrane protein, consists of an extracellular amino-terminal region and two carboxy-terminal transmembrane helices (68). Its distinctive structure enables its involvement in membrane rupture, indicating that it may be pivotal for executing PANoptosis.

**Figure 2:**
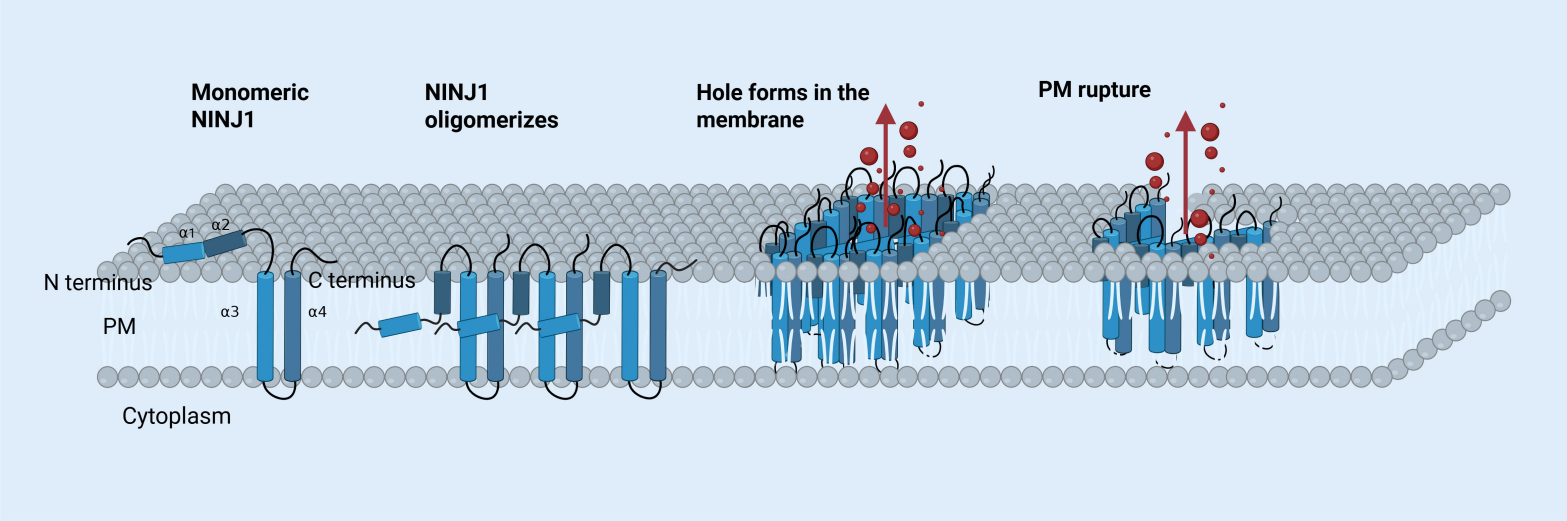
The mechanism of NINJ1-mediated PMR. A monomeric NINJ1 protein consists of four α-helices termed α1, α2, α3, and α4, located between its amino (N) and carboxy (C) termini. In its inactive state, α1 and α2 are positioned outside the plasma membrane. Upon oligomerization, the helices reorient, positioning it against α3 and α4. Oligomerized NINJ1 form a large pore, enabling proteins and inflammatory molecules (shown in red) to exit the cell. The plasma membrane eventually ruptures. [The figure was created using BioRender (https://BioRender.com)].

Kayagaki et al. identified NINJ1 accumulation and oligomerization on the cell surface as key events in plasma membrane rupture (PMR), which ultimately leads to proinflammatory DAMPs releasing (69). This view was validated by Borges et al., who demonstrated that glycine provides cytoprotection against lytic cell death by inhibiting NINJ1 aggregation (70). Kayagaki et al. reported a potent anti-NINJ1 antibody that inhibits NINJ1 oligomerization, thereby preventing PMR and the release of lactate dehydrogenase (LDH) and DAMPs during pyroptosis and apoptosis (71). In 2023, Degen et al. discovered that NINJ1 monomers form oligomers by undergoing conformational changes, creating long, branched filaments in the membrane. Each filament unit consists of four α-helices, with α3 and α4 forming the hydrophobic core, while α1 and α2 integrate into the membrane, facilitating oligomerization mainly through interaction with α3 and α4. This assembly disrupts membrane integrity by exposing hydrophilic regions of α1 and α2, forming pores (72). Han et al. further proposed that NINJ1 is a key executor of PANoptosis, releasing inflammatory molecules independently of other pore-forming proteins such as GSDMD, GSDME, and MLKL (73). **Figure 2** illustrates how NINJ1 leads to cell membrane rupture.

The association between NINJ1 and PCD has garnered great attention in recent years. Han et al. discovered that CASP-8, ASC, and NLRP3 co-precipitated with RIPK3, forming a PANoptosome complex in an *in vivo* heat stress model; As a key molecule in PANoptosis, CASP-8 regulates the oligomerization and spot formation of NINJ1, suggesting the interaction relationship between NINJ1 and PANoptosis (73). Zhou et al. confirmed that NINJ1 is expressed on the platelets’ plasma membrane. And resting platelets showing uniform NINJ1 expression, whereas NINJ1 polymerisation occurs during membrane rupture. This study also found that pretreating platelets with PANoptosis inhibitors significantly reduced the percentage of thrombin-induced NINJ1 oligomerized platelets, indicating that PANoptosis is involved in the rupture progress (74).

In addition to its role in the final stage of cell death, NINJ1 can also contribute to the immune response by mediating leukocyte migration, promoting neutrophil recruitment and infiltration, and regulating the release of DAMPs and inflammatory factors (73, 75, 76). In cerebral ischemic diseases, studies indicate that the endogenous protein NINJ1 inhibits angiogenesis. Conversely, the N-terminal blocking peptide N-NAM (-Pro26 to -Asn37) may enhance angiogenesis by competitively binding to Ninj1 and preventing its homotypic binding (77). Some studies have examined the factors influencing NINJ1-mediated membrane rupture by focusing on its interaction with key proteins involved in PANoptosis, but the relevant literature is limited, more research is needed to clarify the role of NINJ1-mediated membrane rupture in diseases.

## 4 Detection methods for PANoptosis

To demonstrate the occurrence of PANoptosis or assess the impact of a target drug on PANoptosis, researchers must first evaluate cell death. Tweedell et al. proposed two primary approaches for this purpose: i) live-cell imaging using the IncuCyte system to monitor cell death and ii) detection of cell death via the LDH assay (78). Additional methods include the cell-counting kit-8 (CCK8) assay to determine cell viability, terminal deoxynucleotidyl transferase dUTP nick end labelling (TUNEL) staining with quantitative analysis of positive cells, quantitative analysis using Hoechst 33, 342/PI fluorescence staining, and flow cytometry to evaluate cell death (7, 11, 57, 58, 79, 80).

To identify the underlying mechanisms of PANoptosis, many studies primarily use western blotting to assess the expression levels of members involved in various cell death pathways. For instance, the detection of pyroptosis-related proteins such as NLRP3 and increased lysis/total CASP-1 indicates pyroptosis, increased lysis/total CASP3 and a decreased B-cell lymphoma-2 (Bcl-2)/Bcl2-associated x (Bax) ratio indicates apoptosis, and phosphorylation of RIPK3 and MLKL serves as a marker for necroptosis. Inhibition experiments can further demonstrate PANoptosis. While gene knockout models are ideal for studying single-cell death pathways, practical challenges may necessitate the use of specific inhibitors (78). It is needed to prove that knocking out or inhibiting key proteins in a single death pathway is not enough to prevent cell death completely, whereas blocking multiple pathways can entirely inhibit PANoptosis. Besides, we can prove PANoptosis by detecting the PANoptosome. In most studies, PANoptosomes are identified by the co-localization of key proteins in PCDs like ASC, CASP8, and RIPK3 (78). Other techniques for detecting target proteins include IF staining and immunohistochemistry (IHC) (7, 11, 57, 58, 78–80).

## 5 PANoptosis in nervous system diseases

### 5.1 Ischaemia stroke

IS is a disorder of CNS that results in severe neurological deficits and high rates of disability and mortality worldwide (57, 81). Currently, recanalization therapy is the mainstay treatment for cerebral ischaemic stroke. However, restored blood flow can induce oxidative stress (OS), inflammation, and neuron death, resulting in ischemia/reperfusion (I/R) injury, which results in poor prognoses for patients (7, 82).

A growing number of studies are beginning to focus on the impact of PANoptosis on I/R injury. Yan et al. provide theoretical support for the role of PANoptosis in I/R injury using bibliometric analysis and data mining (10). Subsequently, Lan et al. showed the presence of PANoptotic neuronal death under brain I/R injury for the first time, as the expression of vital members of the PANoptosome (AIM2, ZBP1, pyrin) and the essential proteins of pyroptosis, apoptosis, and necroptosis (CASP1, GSDMD, CASP3, CASP8, RIPK1, RIPK3, and MLKL) were significantly enhanced (57).

Some natural compounds exert neuroprotective effects in treating IS (**Table 2**). Curcumin, a diketone compound derived from the traditional medicinal plant turmeric, has potent anti-inflammatory, anti-aging, anticancer, and antimicrobial properties (83, 84). Studies have demonstrated that a combination of curcumin and olfactory mucosa-mesenchymal stem cells (OM-MSCs) exerts neuroprotection against IS by inhibiting neuronal ferroptosis (85). Lan et al. demonstrated that curcumin pretreatment significantly enhanced the neuroprotective efficacy of OM-MSCs against PANoptotic neuronal death in IS. They attributed this effect to the upregulation of miRNA-423-5p in the CUR-OM-MSCs group, which inhibits the NOD2/NF-κB/MAPK signaling pathway and prevents microglia from transforming into the M1 phenotype (57). Besides, the author suggested that the paracrine effect of MSCs (including EVs) may mediate its neuroprotection, but further research is needed to determine if curcumin pretreatment upregulates miRNA-423-5p or other factors by targeting EVs. Esculentoside H (EH), a saponin from the root of Phytolacca esculenta, is used in traditional Chinese medicine for inflammatory diseases. Zhang et al. discovered that EH alleviates PANoptosis after brain I/R injury and protects the blood-brain barrier via the TLE1/PI3K/AKT pathway (7). Future studies should assess the long-term neuroprotective effects, side effects of these treatment, and the optimal concentration and timing of drugs for the best therapeutic outcome.

**Table 2:** Mechanisms underlying the treatment of CNS diseases related to PANoptosis.

Disease	Treatment	Pathway		Mechanism	Reference
IS	Curcumin and OM-MSCs	NF-κB, MAPK		Upregulation of miRNA-423-5p in CUR-OM-MSCs and inhibition of the NOD2/NF-κB/MAPK signalling pathway; Microglia adopt an M2 (anti-inflammatory) phenotype, reducing brain I/R-induced PANoptotic neuronal death.	(57)
	EH	TLE1/PIK3/AKT		Upregulation of TLE1 expression, which downregulates the expression of MMP9 and upregulates the levels of ZO1 and occluding, ultimately protecting the BBB and alleviating PANoptosis	(7)
	m6A-modification of ALKBH5	NA		Downregulation of SNHG3 expression in an m6A-dependent manner Low expression of SNHG3 prevents the formation of the SNHG3-ELAVL1-ZBP1/AIM2 complex, leading to destabilization of ZBP1 and AIM2 mRNAs and subsequent downregulation of these PANoptosis-related genes	(58)
SAE	NA	p38MAPK, ERK		Downregulation of TLR9 inhibits both the p38 MAPK and ERK pathways and finally regulates PANoptosis	(11)
Glaucoma/Injury of retinal neurons	Mdivi-1 and Drp1 siRNA	ERK1/2		Drp1 is involved in mitochondrial fission through post-translational modifications. Inhibition of DRP1 rescues ph-IOP-induced RGC PANoptosis	(59)
	DMHCA	NF-kB		Downregulation of NINJ1 expression and inhibition of NF-κB pathway, which leads to reduced neuroinflammation and apoptosis	(60)
SCI/SCIRI	Zinc ions	Lgals3-Bax		Zinc ions target the Lgals3-Bax axis to protect mitochondria, modulate mitochondrial quality control, and alleviate neuronal PANoptosis in post-SCI neurons.	(61)
	YBX1	NA		Stable ZBP1 mRNA promotes ZBP1 expression, thereby exacerbating ZBP1-mediated apoptosis.	(62)
	Melatonin	NA		Melatonin confers neuroprotection on the spinal cord by preventing PANoptosis.	(63)
	H2S	NA		H2S attenuated SCIRI-induced PANoptosis, microglia/macrophage activation (M1 polarization), and inflammation	(64)

RNA N6-methyladenosine (m6A) modification is a critical epigenetic alteration affecting RNA stability, functionality, and activity (86). Numerous studies have demonstrated that m6A modification has significant potential for the treatment of I/R injury (87–89). Qiu et al. revealed that targeting the ALKBH5/m6A/SNHG3 axis significantly inhibits PANoptosis via the negative regulation of SNHG3 by ALKBH5. Low expression of SNHG3 prevents the formation of the SNHG3-ELAVL1-ZBP1/AIM2 complex, thereby destabilising ZBP1 and AIM2 mRNA, which leads to the downregulation of genes related to PANoptosis, eventually inhibiting the associated inflammatory response (58).

### 5.2 Sepsis-associated encephalopathy

Sepsis is a continuous systemic inflammatory response syndrome resulting from an excessive immune reaction to infection, often resulting in multiple organ dysfunction (90). SAE is a severe complication of sepsis characterised by a decline in mental status and cognitive function (91).

Zhou et al. elucidated the activation of PANoptosis in CLP-induced SAE rats. By detecting death-related proteins and observing the ultrastructure of cortical neurons, the authors confirmed that apoptosis, pyroptosis, and necroptosis occur concurrently in SAE rats. By administering death pathway-specific inhibitors, the authors found a synergistic effect between apoptosis and pyroptosis, thereby modulating necroptosis. Downregulating TLR9 expression can inhibit the p38 MAPK/ERK pathways and suppress PANoptosis, improving survival rates and better outcomes (11). This study highlights how necroptosis activation can inhibit both apoptosis and pyroptosis, revealing the complex relationships among PCDs. Focusing on the interactions among these PCD pathways may become a new approach to clinical intervention.

### 5.3 Glaucoma/injury of retinal neurons

Glaucoma is a diverse group of optic neuropathies characterised by the progressive degeneration of retinal ganglion cells (RGCs), thinning of the retinal nerve fibre layer, and resultant vision loss (92, 93). Pathological intraocular hypertension (IOP) is a risk factor for glaucoma development. It can induce inflammation, retinal OS, I/R injury, and other pathological damage (94). Drp1 is a key protein in mitochondrial division. Zeng et al. found that PANoptosis is involved in Drp1-mediated mitochondria abnormalities. Treatment with Mdivi-1 and Drp1 siRNAs improved OGD/R-induced R28 cell damage and restored mitochondrial function, and Drp1 inhibition also rescued RGC PANoptosis *in vivo* (59). Future research on Drp1-mediated mitochondrial dynamics with PANoptosis, and small molecule inhibitors targeting Drp1 may reveal new therapeutic targets for glaucoma treatment.

Retinal ischaemia-reperfusion injury (RIRI) is a critical pathophysiological basis for various ischaemic retinal diseases, which triggers a complex pathological process involving oxidative stress, apoptosis, necroptosis, vascular damage, and inflammatory responses (95). A literature mining by Yan et al. showed that PANoptosis may be present in neuronal I/R injuries (10). Their experimental results further demonstrated that under the same model conditions and treatment duration, pyroptosis, apoptosis, and necroptosis occurred concurrently after retinal I/R damage in R28 cells induced by OGD/R and retinal I/R damage induced by aHIOP, providing initial evidence for future studies on PANoptosis (96). However, this literature only confirmed PANoptosis occurs after retinal I/R injury but did not explore its regulation, the role of the PANoptosome or other key molecules. Additionally, using three types of inhibitors did not fully prevent cell death from I/R injury, indicating involvement of other regulated cell death or signaling pathways.

### 5.4 Spinal cord injury

SCI is a severe traumatic disorder characterised by extensive neuronal death, significant microglial infiltration and polarisation, and impaired motor function impairment (61).

Single-cell sequencing analysis identified galectin 3 (Lgals3) and Bax as key genes involved in apoptosis. Earlier research has shown zinc’s anti-inflammatory and antiapoptotic properties in treating SCI. Bai et al. found that zinc ions target the Lgals3-Bax axis to protect mitochondria, modulate mitochondrial quality control in neurons after SCI, thereby reducing neuronal PANoptosis. Further research is needed to explore how mitochondrial quality control influences PANoptosis (61). Lou et al. found that YBX1, a multifunctional protein of the RBP family, is highly expressed in neurons following SCI. It could promote ZBP1 expression by stabilizing Zbp1 mRNA, thereby exacerbating apoptosis. In contrast, E3 ubiquitin ligase TRIM56 can inhibit YBX1-mediated PANoptosis by promoting its ubiquitination and degradation (62). Xie et al. revealed that PANoptosis may underlie large-scale neurodegeneration and paraplegia in patients with SCI, and melatonin could provide neuroprotective effects on the spinal cord by preventing PANoptosis (63).

Spinal cord ischaemia-reperfusion injury (SCIRI), a catastrophic surgery complication, can also arise from spinal cord trauma, degeneration, or tumours, leading to sensory and motor dysfunction (97). Xie et al. found that H2S reduces neuronal apoptosis, pyroptosis, and necroptosis by inhibiting microglial polarisation, ultimately alleviating motor dysfunction after SCIRI. These findings indicate the potential application of slow-releasing H2S donors as clinical neuroprotective agents (64). Further research can be conducted on the specific mechanisms by which H2S release inhibits PANoptosis and its effect on microglial polarization.

### 5.5 Alzheimer’s disease

AD is an age-related neurodegenerative disorder. Patients with AD exhibit symptoms such as memory loss, cognitive decline, and visuospatial impairment (98, 99). Previous studies have shown that the primary pathological hallmarks of AD include β-amyloid (Aβ) deposition, abnormal phosphorylation of tau protein, microglial activation, and neuronal loss (98).

Rajesh et al. summarised the role of apoptosis, pyroptosis, and necroptosis in AD and highlighted that several molecules involved in PANoptosis, such as AIM2, CASP8, CASP1, RIPK3, and MLKL, have been identified as key players in neuroinflammation and neurodegenerative diseases, including AD (9). Meng et al. proposed future therapeutic directions targeting Aβ oligomers (AβOs) to activate PANoptosis via mitochondrial dysfunction. They suggest inhibiting the opening of the mitochondrial permeability transition pore (mPTP) to suppress the release of pro-apoptotic factors, mitochondrial DNA (mtDNA), and reactive oxygen species (ROS). Additionally, targeting ROS clearance, repairing mtDNA damage, and enhancing mitochondrial quality control could help alleviate mitochondrial oxidative damage and autophagic defects (100). Zhang et al. constructed a PANscore model based on PANoptosis-related genes using Least Absolute Shrinkage and Selection Operator (LASSO) regression analysis and demonstrated that the model was able to effectively predict the prognosis, helping clinical doctors formulate personalized treatment plans for AD patients (101).

## 6 Discussion

Although pyroptosis, apoptosis, and necroptosis were initially considered three independent PCD pathways, recent research has revealed extensive crosstalk among them (6). The key to understanding PANoptosis lies in understanding the interactions among the three types of PCD pathways. When certain death pathways are prevented, other signalling mechanisms are activated to enhance alternative death pathways, suggesting the existence of a molecular platform capable of simultaneously controlling and regulating various cell death pathways (11, 102). This platform was later defined as PANoptosome. This review summarizes the four defined PANoptosomes, the widely studied NLRP3 and NLRC4 inflammasomes, and their regulation mechanisms. Further research is needed to identify other types and explore the roles of proteins in PANoptosomes and their regulatory pathways. We also discuss the “executor” NINJ1 protein, which acts independently of other pore-forming proteins. It has been greatly studied for its structure and membrane-disrupting mechanism. However, the upstream mechanisms leading to NINJ1 aggregation and its membrane-puncturing effects remain unclear.

PANoptosis-related research primarily focuses on infection and cancer diseases, yet increasing studies have identified PANoptosis in CNS disorders (10). However, most of them have not explored the exact mechanisms that trigger or suppress PANoptosis, remaining primarily at a descriptive level; Although some studies have connected mitochondrial functions to the regulation of PANoptosis, the specific mechanisms underlying remain unclear; Some studies suggest that the activation of necroptosis can suppress both apoptosis and pyroptosis, indicating a non-parallel relationship among the three types of PCD in PANoptosis. However, there is still limited literature exploring the regulatory relationship among these forms of PCD. Further research on the regulatory mechanisms of PANoptosis will provide novel insights into treating CNS disorders.
